# A scale- and orientation-adaptive extension of Local Binary Patterns for texture classification

**DOI:** 10.1016/j.patcog.2015.02.024

**Published:** 2015-08

**Authors:** Sebastian Hegenbart, Andreas Uhl

**Affiliations:** Department of Computer Sciences, University of Salzburg, Jakob-Haringer Strasse 2, 5020 Salzburg, Austria

**Keywords:** LBP, Texture, Classification, Scale, Adaptive, Rotation, Invariant, Scale-space

## Abstract

Local Binary Patterns (LBPs) have been used in a wide range of texture classification scenarios and have proven to provide a highly discriminative feature representation. A major limitation of LBP is its sensitivity to affine transformations. In this work, we present a scale- and rotation-invariant computation of LBP. Rotation-invariance is achieved by explicit alignment of features at the extraction level, using a robust estimate of global orientation. Scale-adapted features are computed in reference to the estimated scale of an image, based on the distribution of scale normalized Laplacian responses in a scale-space representation. Intrinsic-scale-adaption is performed to compute features, independent of the intrinsic texture scale, leading to a significantly increased discriminative power for a large amount of texture classes. In a final step, the rotation- and scale-invariant features are combined in a multi-resolution representation, which improves the classification accuracy in texture classification scenarios with scaling and rotation significantly.

## Highlights

• A scale- and rotation-invariant feature representation based on LBP is presented.
• LBPs are computed adaptively based on the estimated scale of an image.
• An estimate of global orientation is used to align LBP at a high angular resolution.
• Features independent of the intrinsic texture scale increase the adaptability.
• Significant improvements are achieved in scenarios with scaling and rotation.

## 1 Introduction

A major challenge in texture classification is dealing with varying camera-scales and orientations. As a result, research focused on scale- and rotation-invariant feature representations has been a hot topic in the last years. Feature extraction methods providing such invariant representations allow to be categorized into four conceptually different categories.

In a theoretically elegant approach, methods of the first category transform the problem of representing features in a scale- and rotation-invariant manner in the image domain, to a possibly easier, but equivalently invariant representation in a suitable transform domain. Pun et al. [1] utilize the Log-Polar transform to convert scaling and rotation into translation, scale- and rotation-invariant features are then computed using the shift invariant Dual-Tree Complex Wavelet Transform (DT-CWT [2]). Jafari-Khouzani et al. [3] propose a rotation-invariant feature descriptor based on the combination of a Radon transform with the Wavelet transform. A general drawback of this class of methods is that scaling can only be compensated at dyadic steps. As an improvement, Lo et al. [4] use a Double-Dyadic DT-CWT combined with a Discrete Fourier Transform (DFT) to construct scale-invariant feature descriptors at sub-dyadic scales. The periodicity of the DFT is also exploited by Riaz et al. [5] to compute scale-invariant features by compensating the shifts in accumulated Gabor filter responses.

In a more pragmatic approach, methods of the second category achieve scale- and rotation-invariance either explicitly, by a re-arrangement of feature vectors, or implicitly, by selection of suitable transform sub-bands. In general, methods in this class also rely on some sort of image transformation. Lo et al. [6] (using the DT-CWT), Montoya-Zegarra et al. [7] (using the Steerable Pyramid Transform) as well as Han and Ma [8] and Fung and Lam [9] (both relying on Gabor filters responses) are representative approaches of this category. In parallel to the first concept, methods of this class are often limited in the accuracy and amount of compensable scaling and rotation by the nature of the used image transformation.

The obvious, but potentially most devious category, is based on a feature representation with inherent scale- and rotation-invariance. The fractal dimension [10] as measure for the change in texture detail across the scale dimension, is a promising candidate for such a representation. Geometric invariant feature representations based on the temporal series of outputs of pulse coupled neural networks (PCNN) have been used by Ma et al. [11] and Zhan et al. [12]. As a consequence of the inherent scale- and rotation-invariance however, this type of features is likely to have a decreased discriminative power as compared to other feature representations and often requires a generative, model based approach, such as Bag-Of-Words, to be competitive.

The fourth and last category of methods utilizes estimated texture properties to adaptively compute features with the desired invariants. Xu and Chen [13] use geometrical and topological attributes of regions, identified by applying a series of flexible threshold planes. Another large set of methods is based on the response of interest point detectors, such as the Laplacian of Gaussian (LoG [14]), the Harris–Laplace detector [15], Difference of Gaussian (DoG, SIFT [16]), Determinant of Hessian (DoH, SURF [17]) or Wavelet modulus maxima (SIFER [18]) to construct invariant features. Lazebnik et al. [19] apply affine normalization, based on the estimation of local shape and scale at detected interest points, to compute affine invariant features. Hegenbart et al. [20] compute LBP in an affine-adapted neighborhood while Li et al. [21] rely on local responses of the LoG to build a scale-invariant LBP representation. Due to the sparse output of interest point detectors and the stability of selected regions, a feature representation derived from interest points, might not be appropriate for all texture classification scenarios however. Even more, the intrinsic-scale of a large number of textures is inappropriate for a directly adapted computation of discriminative features, due to unsuitably large or small scales. As a consequence, the SIFT, SURF and SIFER feature descriptors are primarily used for tasks in computer vision apart from texture classification. A variation of these methods without scale-selection, based on local descriptors, computed at a dense grid, is generally used for computing features for the classification of textures.

In this work, we present a methodology which combines ideas from the second (alignment of features) and the last category (scale-adaption) to construct a scale- and rotation-invariant LBP feature representation. The method integrates seamlessly into the general computation of LBP, providing a high angular resolution with a fine grained compensation of scaling. Rotation-invariance is achieved by explicit alignment of features at the extraction level, based on a robust global estimate of orientation, using information provided by multi-scale second moment matrices [22]. The distribution of scale normalized Laplacian responses, in a scale-space representation of an image, allows a reliable estimation of the global image scale, which is used for a scale-adaptive feature computation. Based on the estimation of the global scale, intrinsic-scale-adaption is applied to compute features independent of the intrinsic texture scale. This assures the use of suitable LBP-radii, increasing the discriminative power of the feature representation significantly for a large amount of texture classes. In a final step, the rotation- and scale-invariant features are combined in a multi-resolution representation to further improve the discriminative power.

### 1.1 Limitations of LBP with image scaling and rotation

The Local Binary Pattern method [23] represents textures as the joint distribution of underlying microstructures, modeled via intensity differences in a pixel neighborhood. Such a neighborhood is defined in relation to a center pixel at position (*x*,*y*) as a tuple of *n* equidistant points on a circle with a fixed radius *r*. The position of neighbor number *k* is computed as

(1) $$\eta^{r,n}(k;x,y)={\left(\begin{array}{c} x+r\cos(\frac{2\pi k}{n})\\ y-r\sin(\frac{2\pi k}{n}) \end{array}\right)}^{T}.$$

A weighted sum, representing the pixel neighborhood, is computed and interpreted as binary label, based on a sign function sgn(*x*) mapping to 1 if x≥0 and 0 else. For a position (*x*,*y*) in an image, the standard LBP, based on *n* neighbors and radius *r* is computed as

(2) $${\mathrm{LBP}}^{r,n}(x,y)=\overset{n-1}{\underset{k=0}{\sum }}2^{k}\;\mathrm{sgn}(I(\eta^{r,n}(k;x,y))-I(x,y)).$$

Finally, the distribution of patterns is represented by a histogram, which is then used, in conjunction with a meaningful distance function, as an LBP feature.

The LBP feature representation has been used in a wide range of texture classification scenarios and has proven to be highly discriminative. A restriction of LBP however, is its sensitivity to affine transformations. As a consequence of the fixed-scale radius and the fixed sampling area dimension of the pixel neighborhood, the locally computed patterns implicitly encode the underlying micro structures of a texture at a scale directly related to the camera-scale of an image. As a result, the LBP feature representation is unable to compensate for different camera-scales. Even more, a rotation of an image is reflected as a circular shift in the individual patterns, which affects the distribution of patterns in a non-linear fashion. As a consequence, the standard LBP feature representation requires either an implicit or explicit alignment of patterns, which is generally done at the encoding level, to compensate for image rotations.

A widely used rotation-invariant encoding of LBP is based on the work of Ojala et al. [24]. The authors construct a rotation-invariant representation at the encoding level by implicit alignment of patterns, representing each individual pattern as the minimal decimal interpretation of all possible bitwise circular shifts of that specific pattern. A major limitation of encoding level based approaches is the highly limited angular resolution. As a consequence, Ojala et al. [24] suggest to combine their rotation-invariant encoding with uniform LBP. This combination however, leads to an even smaller number of individual patterns and a possibly decreased discriminative power of the feature representation. In the same work, the authors propose a multi-resolution representation, which improves the discriminative power of the features, by adding the capability of describing underlying micro structures at multiple scales. The multi-resolution representation however lacks a scale-selection mechanism and is therefore unable to compensate for image scaling.

Li et al. [21] were the first to compute scale-adapted LBP, based on the estimation of local texture scale. The authors use a direct mapping from the estimated local texture scale (in terms of the scale-space) to compute scale-adapted LBP-radii. Rotation-invariance is achieved, based on the methodology proposed by Guo et al. [25], estimating a global orientation on the basis of the computed LBP distribution and using bit alignment on a sub-uniform basis. Unfortunately, using the estimated local image scale as LBP-radius, significantly reduces the reliability of the method. This is a result of computing the features in dependence of the intrinsic texture scale, which is inappropriate for a large number of texture classes (in particular natural textures), due to either very large LBP-radii (low discriminative power) or very tiny LBP-radii (limited possibility of scale-adaption).

The proposed scale- and orientation-adaptive (SOA)-LBP, based on prior work [26,27], addresses these limitations. The low angular resolution of encoding level based rotation-invariant representations is significantly improved by alignment of patterns at the extraction level, using a robust estimate of global texture orientation. The reliability of the feature representation is greatly enhanced by the means of intrinsic-scale-adaption, allowing the computation of highly discriminative features, independent of a texture׳s intrinsic-scale.

## 2 Scale-adaptive Local Binary Patterns

We compute a scale-invariant representation of LBP by appropriate selection of LBP-radii (Section 2.2), based on a global estimate for image scale (Section 2.1). To compensate for the changed spatial extent of image structures due to scaling, we perform Gaussian low-pass filtering in reference to the corresponding scale-adapted LBP-radius, to sample neighbors at the correct scale (Section 2.3).

### 2.1 Estimation of the global image scale

We estimate the global scale of an image utilizing the distribution of scale-normalized Laplacian responses in scale-space. Let $f:R^{2}\mapsto R$ represent a continuous signal, then the scale-space representation, parametrized in terms of the standard deviation of the Gaussian, $L:R^{2}\times R_{+}\mapsto R$ is defined by

(3) L(·;σ)=g(·;σ)⁎f,

with initial condition L(·;0)=f. We denote $\sigma \in R_{+}$ as the scale parameter (the standard deviation of the Gaussian function *g*) and “⁎” represents a convolution operation. The scale-space family *L* is the solution to the diffusion equation

(4) $$\partial_{\sigma }L=\sigma (\frac{\partial^{2}L}{\partial x^{2}}+\frac{\partial^{2}L}{\partial y^{2}})=\sigma \Delta L.$$

We construct the scale-space using an exponential spacing of scales $\sigma_{i}=c{\sqrt{2}}^{k_{i}},k_{i}\in \{-4,-3.75,\ldots ,7.75,8\}$ and *c*=2.1214. The value of *c* acts as a scaling factor and was initially chosen such that the center scale of the representation corresponds to the LBP-radius 3. We later added a set of larger scales to accommodate for the large intrinsic-scales of natural textures. By using an exponential spacing, we provide a fine grained estimation at small scales and still cover a considerable amount of large scales. Note that as a result of the Gaussian filtering for computing suitable sampling support areas, estimation errors at large scales are not as significant as errors at small scales. The used scale-space parametrization provides a solid foundation for estimating scales in a large number of scenarios. A parametrization specifically optimized for a given problem could potentially improve the accuracy in some cases however.

**Fig. 1:**
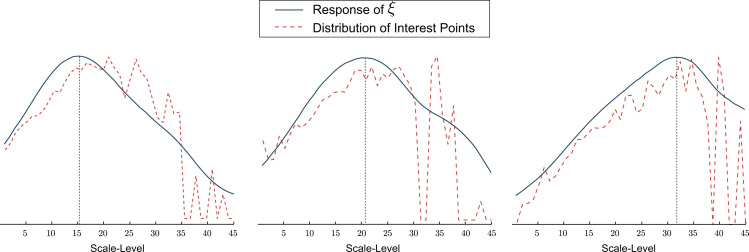
Normalized response of *ξ* compared to the normalized response distribution of scale-space extrema.

As a consequence of the sparse output of interest point detectors, scale estimation based on such scale-space extrema has shown to be unreliable for a large number texture classes. Fig. 1 illustrates this by comparing the response distribution of scale-space extrema with the proposed scale estimation function *ξ*. It can be observed that the sparse nature of interest points significantly limits the reliability of the scale estimation.

**Fig. 2:**
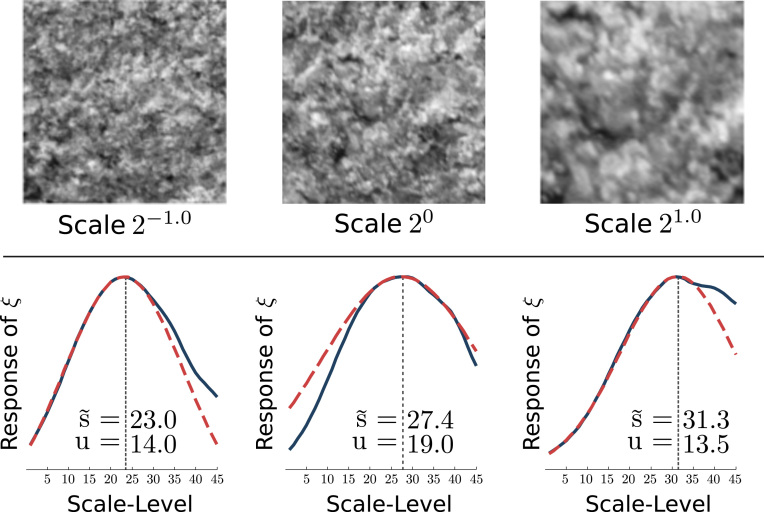
Estimated scales-levels $\overset{˜}{s}$ with uncertainty *u* for a texture at three camera-scales. (For interpretation of the references to color in this figure caption, the reader is referred to the web version of this paper.)

We therefore use the distribution of the responses of scale-normalized Laplacians in the scale-space representation of an image *I*, ($\sigma^{2}|\Delta L(\cdot ;\sigma )|$, denoted as $\overset{¯}{\Delta }I(\cdot ;\sigma )$), computed at all scales in the scale-space, to estimate a global image scale. The scale estimation function *ξ* is

(5) $$\xi (\sigma_{i})=\underset{z}{\sum }\overset{¯}{\Delta }I(z;\sigma_{i}),$$

for $z\in R^{2}$ corresponding to a Cartesian coordinate on the pixel grid and *σ*_*i*_ denoting a specific scale-level in the scale-space. To determine the global scale of an image, the first local maximum of *ξ* is searched, which is then used as seed point for a least-squares Gaussian fit. By using the first local maximum we are capable of consistently estimating the scale of textures exhibiting more than a single dominant global scale. The quality of the estimation is improved by using only data points within a certain offset from the seed point. We use 10 percent of the number of scale-levels in the scale-space as positive and negative offset from the estimated first local maximum to fit the Gaussian function. This value was found during development of the method and has proven to be very stable for various image datasets. The mean value $\overset{˜}{s}$ of the fitted Gaussian function is interpreted as the dominant level in scale-space. The standard deviation *u* of the fitted Gaussian is used as uncertainty of the estimation. For a given dominant scale-level in scale-space $\overset{˜}{s_{i}}$, the spatial scale *s*_*i*_ corresponds to the scale parameter *σ*_*i*_ in $L(\cdot ;\sigma_{i})$ (the extent of a spatial structure at scale *s*_*i*_ is $\sigma_{i}\sqrt{2}$). Fig. 2 illustrates the determination of a global scale by fitting a Gaussian function (dashed red line) to the scale estimation response function *ξ* (solid blue line).

The scale estimation method is reliable for the majority of evaluated images but fails completely for a small fraction (approximately 3%). We identify a failed scale estimation by evaluating the uncertainty *u*. In our implementation, the scale estimation is considered as failed if *u*, normalized by the number of scale-levels, is greater than a certain threshold *t*. In such a case, scale-adapted radii cannot be computed reliably. We therefore fall back to a default, computing the standard LBP with a fixed radius. The value of *t* was chosen as 20/n for *n* scale-levels (in our case *t*=0.4082). The specific value for the threshold was found during development and was consequently used across all experiments in this work. Multiple experimental results suggest that this threshold is robust and should generalize well for a large set of different scenarios.

**Fig. 3:**
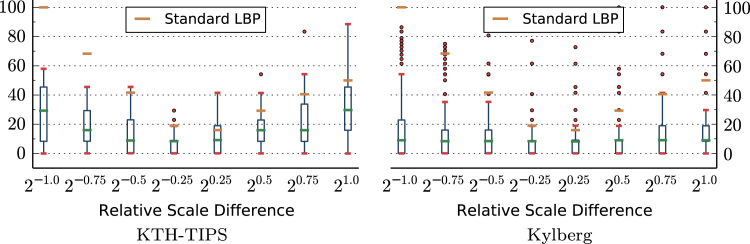
Relative error (in percent) of scale-adapted LBP-radii.

We evaluated the accuracy of the scale estimation for computing scale-adapted LBP-radii, by estimating the global scale of all images in the KTH-TIPS and Kylberg image sets (see Section 5.1) at all 9 scales. Images at the default training scale ($2^{0}$) were then used as reference for computing the relative error of scale-adapted LBP-radii compared to the theoretically optimally scale-adapted radius. Fig. 3 presents the relative error (in percent) of scale-adapted LBP-radii, compared to the error of a fixed-scale LBP radius.

The results show that the relative errors of scale-adapted LBP-radii are significantly smaller as compared to the fixed-scale LBP-radius. This indicates that the computation of scale-adapted patterns should improve the scale-invariance of the feature representation. Note the general asymmetry of the relative error, which can be observed for the fixed-scale LBP radii.

### 2.2 Intrinsic-scale-adaption of the LBP-radius

The visualized scale of an image in the pixel domain is a function of the camera-scale, which is dependent on intrinsic- and extrinsic-camera parameters such as the focal length, the camera-distance, the image sensor dimensions and resolution, as well as the intrinsic-scale of the texture. The intrinsic-scale of a texture can be interpreted as the spatial extent of its dominant structures. The estimation of the intrinsic-scale is only possible with full knowledge of all camera-parameters. Responses of the scale-normalized LoG attain a maximum if its zeros are aligned with a circular shaped image structure. As a consequence, scales estimated based on the LoG correlate strongly with the visualized scale of the dominant circular shaped structures of a texture. The estimated scale of a texture (using our approach) is therefore highly related to the underlying intrinsic-scale.

Considering that the spatial extent of a circular structure is determined by its diameter, we model the visualized scale of an object using a two dimensional pinhole camera model. For an object with intrinsic-scale ι at distance *u* to the lens, the scale of the visualized object on the image sensor of a camera with focal length *f* is given as

(6) $$s=\frac{\iota f}{u}$$

The effective scale of the visualized object in pixels is only dependent on the image sensor format and resolution, which are intrinsic camera-parameters.

**Fig. 4:**
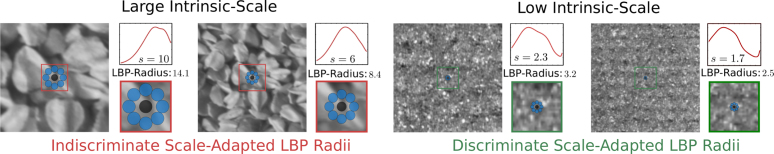
Impact of intrinsic-scales on scale-adapted LBP-radii.

An entire category of methods utilizing local texture properties to compute adapted, invariant features (such as Affine Invariant Regions [19] or Li-LBP [21]) are affected negatively by the large variety of intrinsic-scales across texture classes. This is a consequence of using the estimated scale (as combination of the intrinsic- and camera-scale) directly to compute adapted features. Due to unsuitably large or tiny intrinsic-scales for a considerable amount of texture classes, the estimated scales are likely to be inappropriate for computing scale-adapted features. Fig. 4 illustrates how inappropriate intrinsic-scales potentially lead to indiscriminative (too large) LBP-radii after scale-adaption.

In this work, we propose a method to compute scale-adaptive LBP at suitable and highly discriminative scales by the means of intrinsic-scale-adaption, which allows scale-adaption based on the camera-scale without actual knowledge of the intrinsic-scale. Considering the quotient of two estimated image scales, either the intrinsic-scales cancel each other out (the images are from the same texture class, hence $\iota_{1}\approx \iota_{2}=\iota$)

(7) $$\frac{s_{1}}{s_{2}}=\frac{\iota f}{u_{1}}\frac{u_{2}}{\iota f}\approx \frac{u_{2}}{u_{1}}$$

and the quotient is therefore in terms of the camera-scale, or the intrinsic-scales do not match (images are from different texture classes) and the quotient is basically random. By explicit computation of scale-adapted patterns, based on the quotient between the estimated scale of an image and a trained-base-scale, we are able to adapt for unsuitable intrinsic-scales implicitly. Note that this approach assumes that the used cameras have comparable focal lengths, sensor formats and resolutions and the intrinsic-scales within texture classes have moderate variance.

A trained-base-scale, acting as reference for the computation of intrinsic-scale-adapted patterns, is assigned to each texture class in the training data. In particular, we estimate the scales of each image in the training data and use the median of all estimated scales within a texture class as the trained-base-scale of that class. The scale-adapted LBP-radius used for an image with an estimated scale *s*, in reference to the trained-base-scale ${\overset{¯}{s}}_{l}$ of texture class *l*, is then computed as

(8) $$\lambda (s,l,\rho )=\rho \frac{s}{\overset{¯}{s_{l}}}.$$

We define *ρ* (referred to as base-radius) as the LBP-radius used at the trained-base-scale $\overset{¯}{s_{l}}$. As a trade-off between discriminative power of the representation and the ability of adapting to a large variety of camera-scales, we set ρ=3 as default. This allows for highly discriminative patterns in the case of small relative scale differences and allows to compensate scale differences of up to a factor of 3. Note the linearity of *λ* is a necessary property for scale-invariance. By computing LBP-radii as a function of the quotient of the estimated image scale and a trained-base-scale, the scale-adaptive representation is independent of the intrinsic-scale of the texture. As a consequence, highly discriminative features at suitable LBP-radii can be computed for a much larger set of texture classes.

Our experiments have shown that scale-adapted LBP computed in reference to a wrong trained-base-scale (the wrong texture class), exhibit appropriately the same intra-class variability as compared to the inter-class variability of features computed at matching trained-base-scales (the correct texture class). This is a direct result of the basically random LBP-radii used to compute scale-adapted patterns in such a case. As a consequence, we distinguish between the computation of training features and evaluation features.

The correct class is obviously known for images in the training data as part of the available ground-truth. We therefore compute training features only in relation to the trained-base-scale of the class of each specific image. Concerning images for evaluation, the class labels are unknown. In this case, features are computed in reference to each texture class, with the corresponding trained-base-scale. During classification, only features computed in reference to the same trained-base-scale are compared (see Section 4).

By using this approach we assure that features for training will be computed at suitable discriminative scales, close to the base-radius *ρ* for a majority of images in the training data. Features for evaluation, computed in reference to the correct trained-base-scale (the same class), benefit from intrinsic-scale-adaption, while evaluation features computed in reference to the trained-base-scale of a different texture class are uninformative due to inappropriate (random) LBP-radii and are insignificant for a later classification.

### 2.3 Adaptive sampling support area dimension

Scaling of an image changes the spatial extent of textural structures. Therefore the number of pixels covering structural information changes as well. As a consequence, the size of the sampling support area in the LBP neighborhood has to be adapted accordingly. By applying a Gaussian filter, each pixel in the image implicitly encodes information about a circular neighborhood of appropriate spatial scale. The radius of the Gaussian filter for a texture at estimated scale *s* in relation to a texture class *l* using base-radius *ρ* is computed as

(9) $$g_{r}=\frac{\lambda (s,l,\rho )\pi }{n},$$

for *n* defining the number of LBP-neighbors. The Gaussian filter coefficients are then computed such that *P* percent of the mass of the Gaussian function is covered within the interval $\left[-g_{r};g_{r}\right]$ (the kernel is truncated outside the interval limits):

$$\int_{-g_{r}}^{g_{r}}e^{-(x^{2}/2\sigma_{g}^{2})}\;\mathrm{dx}=P\int_{-\infty }^{\infty }e^{-(x^{2}/2\sigma_{g}^{2})}\;\mathrm{dx}$$

$$2\int_{0}^{g_{r}}e^{-(x^{2}/2\sigma_{g}^{2})}\;\mathrm{dx}=P\sigma_{g}\sqrt{2\pi }$$

(10) $$\sigma_{g}=\frac{g_{r}}{\sqrt{2}\;{\mathrm{erf}}^{-1}(P)}.$$

We chose *P* to be 0.99 which corresponds to 99% of the mass of the Gaussian function, a value that proved to be robust in a large number of classification scenarios. As the sampling of a Gaussian function with very few sampling points potentially leads to a significant error, we use the error function (erf) using a numerical approach based on Abramowitz and Stegun [28] to improve the stability of sampling the one dimensional Gaussian filters centered at 0

(11) $$G(x;\sigma_{g})=\frac{-\mathrm{erf}(\frac{x-0.5}{\sigma_{g}})-\mathrm{erf}(\frac{x+0.5}{\sigma_{g}})}{2},$$

which are then used in a separable convolution with the analyzed image.

### 2.4 Computation of scale-adapted Local Binary Patterns

The position of LBP-neighbor *k*, in a scale-adapted computation, in reference to texture class *l* and an estimated global image scale *s*, using base-radius *ρ* with *n* neighbors is computed as

(12) $$\eta_{l,s}^{\rho ,n}(k;x,y)={\left(\begin{array}{c} x+\lambda (s,l,\rho )\cos(\frac{2\pi k}{n})\\ y-\lambda (s,l,\rho )\sin(\frac{2\pi k}{n}) \end{array}\right)}^{T}.$$

A Gaussian filter *G* with the appropriate standard deviation *σ*_*g*_ (see Eq. (10)) is used to sample neighbors at the correctly adapted spatial scale. Finally, the scale-adapted LBP is computed at position (*x*,*y*) with neighborhood $\eta_{l,s}^{\rho ,n}$ based on the convolution of image *I* with *G*, $\left(I_{g}=I\;⁎\;G\right)$, as

(13) $$\mathrm{SA}-{\mathrm{LBP}}_{l,s}^{\rho ,n}(x,y)=\overset{n-1}{\underset{k=0}{\sum }}2^{k}\;\mathrm{sgn}(I_{g}(\eta_{l,s}^{\rho ,n}(k;x,y))-I_{g}(x,y)).$$

The histogram of patterns computed in reference to the trained-base-scale of texture class *l* is denoted as *H*_*l*_ and added to the SOA-LBP meta-descriptor of the specific image (see Section 4).

## 3 Orientation-adaptive LBP

To compensate for the non-linear changes of the LBP distribution caused by a rotation of an image, an explicit or implicit alignment of patterns is required. This is generally performed at the encoding level, leading to a low angular resolution. To improve the angular resolution, we perform pattern alignment at the extraction level, which integrates naturally with the scale-adaptive computation of LBP and is based on an estimate of global image orientation.

### 3.1 Estimation of the global image orientation

A main requirement on the orientation estimation in the context of scale-adaptive LBP, is robustness to varying image scales. We therefore utilize multi-scale second-moment-matrices (SMM [22]), computed at the global scale of an image, to estimate a global image orientation. The SMM summarizes the predominant directions of the gradient in a specific area of an image. In contrast to the single-scale SMM, the multi-scale SMM is defined over two scale parameters, the local scale *σ*_*i*_ as well as the integration scale *i*. This allows us to estimate the shape of visual structures at appropriate scales, as detected by the scale-estimation algorithm. The integration scale parameter is chosen in relation to the local scale (we use $i=\sqrt{2}\sigma_{i}$). The local scale parameter is selected as the global scale of the image, using the method described in Section 2.1. The multi-scale SMM of an image at location $z\in R^{2}$ is then computed as

(14) $$\mu (z;\sigma_{i},i)=\int_{\xi \in R^{2}}(\nabla I)(z-\xi ;\sigma_{i}){\left(\nabla I\right)}^{T}(z-\xi ;\sigma_{i})g(\xi ;i)\;d\xi .$$

We denote $\left(\nabla I)(z;\sigma_{i}\right)$ as the gradient of the scale-space representation of image *I* at scale *σ*_*i*_ and position *z*. An important property of SMMs in general is positive definiteness. The two (non-negative) eigenvalues of an SMM correspond to the length of the axes of an ellipse (up to some constant factor). The orientation of the eigenvectors correspond to the orientation of the dominant gradient and the orientation perpendicular to the dominant gradient respectively.

To estimate the global orientation of an image *I*, we compute multi-scale SMMs at a dense grid, corresponding to pixel locations $z\in R^{2}$. The orientation at a specific location is determined as the angle between the major axis of the ellipse and the vertical axis of the coordinate system (the axes of the image). Due to the ambiguous orientation of the ellipse, we treat all angles modulus *π*. Hence, the estimated orientation is unambiguous in [0;π]. We then estimate the global orientation of an image, based on the distribution of local orientations, computed at all coordinates of the sampled grid.

In parallel to the scale estimation method described in Section 2.1, this is done by fitting a Gaussian function to the distribution of local orientations in a least-squares optimization. To improve the accuracy of the estimation, we remove data points with an offset greater than ±15° (a robust, empirically found value that was used successfully on various datasets) from the maximum of the distribution, prior to the fitting process. Finally, the average value of the Gaussian is interpreted as the global orientation, which is used to align the sampling points of the orientation-adaptive LBP.

**Fig. 5:**
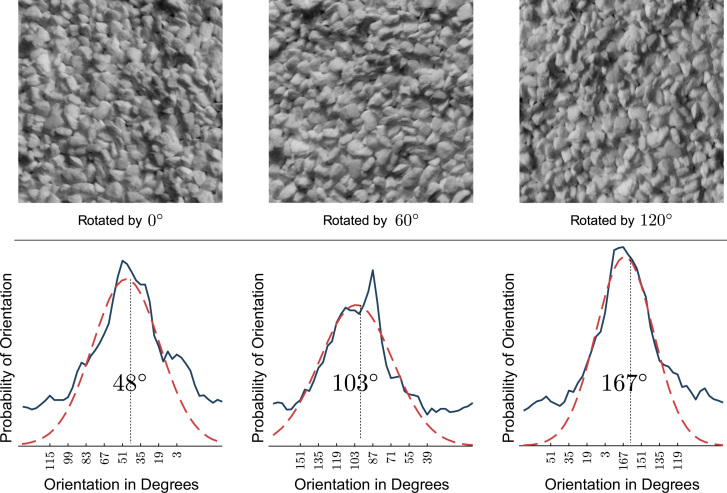
Estimated orientations for a texture at three orientations. (For interpretation of the references to color in this figure caption, the reader is referred to the web version of this paper.)

Fig. 5 illustrates the determination of the global orientation from the local orientation distribution. The dashed red line represents the Gaussian function fitted to the distributions of local orientations (solid blue line) of an image at three different orientations. The numbers centered at each figure present the estimated global orientation of each image.

**Fig. 6:**
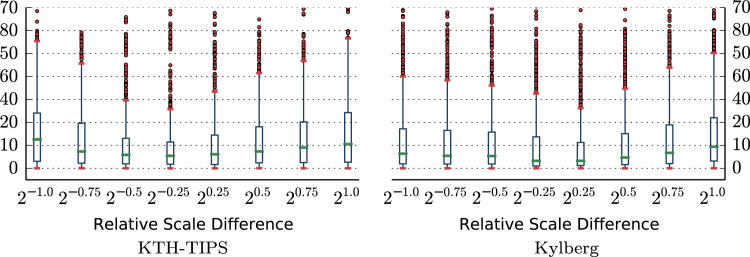
Absolute errors (in degrees) of the orientation estimation.

To evaluate the accuracy of the orientation estimation method, we computed the absolute error of the estimated orientations (Fig. 6) between a reference image at the default training scale ($2^{0}$) and the same image at a different scale and random rotation between 30° and 330° in steps of 30°. The error was evaluated from 891 (81×11) random samples at 8 relative scales using the KTH-TIPS as well as the Kylberg image sets (see Section 5.1).

The results indicate that the orientation estimation method is robust in respect of image scaling. We see across all scales that the medians of the absolute errors are within a range of 5–10°. Experiments have shown that the standard multi-resolution LBP representation can compensate alignment differences of up to 10°, but fails for orientation differences above. In order to improve the orientation-adaptive representation we apply an error compensation technique based on the accumulation of LBP distributions at multiple orientations.

### 3.2 Orientation estimation error compensation

**Fig. 7:**
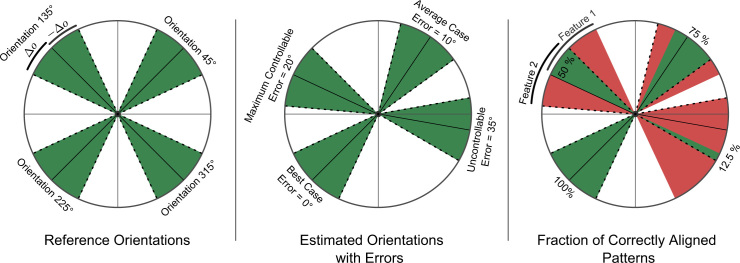
Orientation estimation error compensation using accumulated pattern distributions.

We found that a distribution of LBP with a small amount of misaligned patterns (a systematic error) will be dominated by the majority of correctly aligned patterns. As a consequence, we accumulate the distribution of LBP based on multiple orientations within an interval of ±Δo=20° of the estimated global orientation *o*. Experiments show that by using this approach an estimated error of up to 20° can be compensated without a significant loss of discriminative power of the feature representation. Fig. 7 illustrates this error compensation technique.

To improve the reliability of this scheme, we use thresholding to avoid heavy fluctuation of bits due do interpolation artifacts. The modified sign function sgn(*x*) used in computing the individual patterns therefore requires x≥T to map to 1. The value of *T* is selected adaptively based on the Gaussian filtered image *I*_*g*_, to accommodate for the adapted image properties, as the square root of the standard deviation of all pixel values in *I*_*g*_.

### 3.3 Computation of orientation- and scale-adaptive LBP (SOA-LBP)

To compute SOA-LBP in reference to a texture class *l*, estimated global image scale *s*, global orientation *o*, base-radius *ρ* and *n* neighbors, the position of neighbor *k* is adapted as

(15) $$\eta_{l,s,o}^{\rho ,n}(k;x,y)={\left(\begin{array}{c} x+\lambda (s,l,\rho )\cos(o+\frac{2\pi k}{n})\\ y-\lambda (s,l,\rho )\sin(o+\frac{2\pi k}{n}) \end{array}\right)}^{T}.$$

**Fig. 8:**
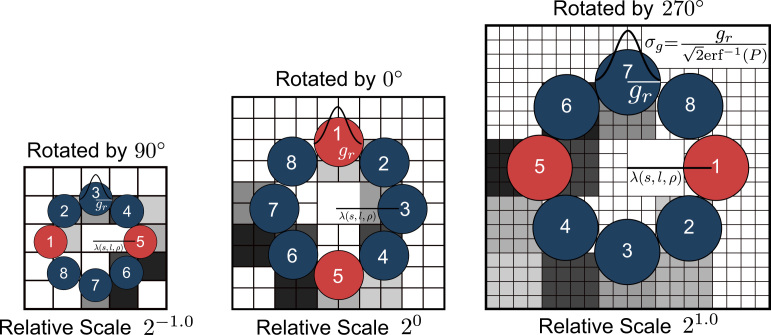
Schematic computation of scale- and orientation-adaptive LBP. (For interpretation of the references to color in this figure caption, the reader is referred to the web version of this paper.)

The actual computation of LBP then follows the scheme of the scale-adaptive LBP as depicted in Section 2.4. To accommodate for the ambiguous orientation of multi-scale SMMs, we compute two patterns with initial sample positions at *o* and o+π respectively. Fig. 8 illustrates the computation of scale- and orientation-adaptive LBP schematically. The red sampling points indicate the initial sample positions.

## 4 SOA-LBP in a multi-resolution feature representation

The computation of multiple LBP-features (histograms) per image, each in reference to an individual trained-base-scale, requires the construction of a meta-feature-representation for classification. We abstract the set of computed LBP-features per image as a single SOA-LBP meta-descriptor and define a meaningful distance function between a pair of such descriptors. A meaningful distance exists only between LBP-features computed in reference to the same trained-base-scale. As a consequence, we define the distance between LBP-features computed at different trained-base-scales as ∞. Experimentation has shown that LBP-features computed at incorrectly adapted scales generally yield a significantly higher intra-class variability as compared to LBP-features computed at correctly adapted scales. The distance between two meta-descriptors is therefore defined as the minimum distance between all pairs of LBP-features abstracted by the descriptors. For two SOA-LBP meta-descriptors $M_{1}$ and $M_{2}$, both representing a set of LBP-features, each computed individually in reference to a texture class in the training data $\left\{H_{1},\ldots ,H_{n}\right\}$, the distance is defined as

(16) $$D(M_{1},M_{2})=\min\{d(H_{l},H_{k})|H_{l}\in M_{1}\wedge H_{k}\in M_{2}\},$$

with

(17) $$d(H_{l},H_{k})=\{\begin{array}{cc} 1-\overset{N}{\underset{i=1}{\sum }}\min(H_{l}(i),H_{k}(i)) & \mathrm{if}\;l=k\\ \infty & \mathrm{if}\;l\neq k. \end{array}$$

In our implementation the histogram-intersection is used as a measure for similarity. A notable drawback of using the meta-descriptor abstraction is that it does not easily integrate with all classification methodologies. We therefore restrict the experimentation in this work to a classification method that allows for a straight forward integration (a standard *k*-nearest neighbors classifier).

Algorithm 1 Selection of valid multi-resolution feature subsets.

**Table:** 

**Data:** Let *H*^1^_*l*_ and *H*^2^_*l*_ be the sets of multi-resolution LBP-features
(histograms) computed in reference to texture class *l* at the base-radii
$\rho =\{\rho_{1},\rho_{2},\rho_{3}\}$ for two images with estimated scales *s*_1_ and *s*_2_.
$H_{l}^{1}=\{h_{l,\rho_{1}}^{1},h_{l,\rho_{2}}^{1},h_{l,\rho_{3}}^{1}\}$ and $H_{l}^{2}=\{h_{l,\rho_{1}}^{2},h_{l,\rho_{2}}^{2},h_{l,\rho_{3}}^{2}\}$
**Result:** Valid subsets *V*_1_,*V*_2_ of features from *H*^1^_*l*_ and *H*^2^_*l*_.
$V_{1}=H_{l}^{1}$ and $V_{2}=H_{l}^{2}$
**foreach**$\rho_{i}\in \rho$**do**
$|\begin{array}{c} r_{1}=\lambda (s_{1},l,\rho_{i})//\mathrm{intrinsic}-\mathrm{scale}-\mathrm{adapted}\;\mathrm{LBP}-\mathrm{radius}\;\mathrm{of}\;h_{l,\rho_{i}}^{1}\\ r_{2}=\lambda (s_{2},l,\rho_{i})//\mathrm{intrinsic}-\mathrm{scale}-\mathrm{adapted}\;\mathrm{LBP}-\mathrm{radius}\;\mathrm{of}\;h_{l,\rho_{i}}^{2}\\ \mathrm{if}\;\min(r_{1},r_{2})<1\;\mathrm{or}\\ \;\max(r_{1},r_{2})>5.44\;\mathrm{or}\\ \;\max(r_{1},r_{2})/\min(r_{1},r_{2})>3\;\mathrm{then}\\ |\begin{array}{c} V_{1}=V_{1}\backslash h_{l,\rho_{i}}^{1}\\ V_{2}=V_{2}\backslash h_{l,\rho_{i}}^{2} \end{array}\\ \mathrm{end} \end{array}$
**end**

Ojala et al. [24] suggest to compute multiple LBP-features, each at separate fixed LBP-radii, to improve the discriminative power of the feature representation. Multi-resolution LBP-features are then created from a set of standard LBP-features by concatenation.

We combine the rotation- and scale-invariant SOA-LBP in a multi-resolution feature representation, to improve the general discriminative power, by reducing the required amount of low-pass filtering for adapting the sampling area and adding the capability of describing underlying microstructures at multiple scales.

Experimental results on various image texture sets suggest that the discriminative power of the multi-scale LBP representation starts to decrease at scales larger than LBP-scale 3 (this corresponds to a radius larger than 5.44 pixels). We therefore consider radii within the interval [1;5.44] to be the most discriminative. To compute scale-adaptive patterns at multiple resolutions, we use a set of distinct base-radii for intrinsic-scale adaption $\rho =\{\rho_{1},\rho_{2},\rho_{3}\}=\{1.5,3,4.5\}$, instead of relying on a single base-radius. Hence, a multi-resolution SOA-LBP representation computed in reference to texture class *l* consists of the set of SOA-LBP-features computed at each of the base-radii and is denoted as $H_{l}=\{h_{l,\rho_{1}},h_{l,\rho_{2}},h_{l,\rho_{3}}\}$.

The specific values for the base radii were chosen to guarantee a high discriminative feature representation for texture image at small scale differences and a minimum amount of required low-pass filtering during scale-adaption for textures at larger scale differences. The values were chosen to augment the default base-radius of 3 in equal steps within the interval of the most discriminative radii.

Considering the small radius $\rho_{1}=1.5$ as well as the large radius $\rho_{3}=4.5$ it is likely that either the lower- or the upper-bound on discriminative LBP-radii is violated for a considerable amount of images, which effectively reduces the discriminative power of the multi-resolution representation. We therefore adaptively select the best subset of SOA-LBP-features for constructing the multi-resolution representation during each computation of the distance between two SOA-LBP meta-descriptors (see Algorithm 1).

Once the best subset of SOA-LBP-features is identified for a pair of meta-descriptors, the final multi-resolution representation is constructed by simple concatenation of the normalized histograms. Note that as a consequence of considerably different intrinsic-scales, or a failed scale estimation, the possibility of $V_{1}=V_{2}=\emptyset$ exists. In such a case, it is likely that the two SOA-LBP-features represent different texture classes. We consider such a pair of features as incomparable in a scale-adaptive sense and define the distance as ∞.

## 5 Experiments

We evaluate the proposed SOA-LBP in reference to a set of scale- and orientation-invariant methods, representative for all categories discussed in Section 1. To assess the reliability of the intrinsic-scale-adaption for a large number of textures, we rely on three different images sets for experimentation. We specifically study the scale-invariance properties (Section 5.4) as well as the effects of combined scaling and rotation (Section 5.5). We finally present a runtime performance analysis of the SOA-LBP (Section 5.6) in relation to the compared methods.

### 5.1 Image data

We perform the experimentation on three image sets with appropriate characteristics. Table 1 summarizes the most important information about the used data.

*CURET*. The CURET image set contains data with different viewing and illumination conditions. In a four-class classification scenario, textures at two different scales are available as 200×200 pixel images. The scale difference of the textures is reported to be approximately 1.7. As a consequence of the significant amount of signal noise in the CURET data, this image set provides an interesting opportunity to evaluate the effects of noise on the proposed method.

*KTH-TIPS*. The KTH-TIPS [29] image set consists of images from 10 different materials captured at 9 individual relative scales between $2^{-1.0}$ and $2^{1.0}$ with 9 samples per material. Due to the dimension of the original images of material “cracker” (the texture would only fill half of the images at certain scales), we could not use this class for simulating rotations and consequently removed the class in all experiments, leading to a classification scenario with only 9 classes. Sub-images of size 128×128 pixels were extracted from the center of each image to be consistent with the orientation evaluation experiments.

*Kylberg*. The Kylberg texture set [30] consists of 28 materials captured at a single camera-scale. The data set contains rotated versions of each image at 30 degree steps within a range of 0–330°. The large image size (576×576 pixels each) allows us to simulate signal scaling without relying on up-sampling, which leads to a reduced amount of unwanted interpolation artifacts. We simulated scaling to match the scales of the KTH-TIPS set such that the scale of the original images is interpreted as the maximum scale $2^{1.0}$ (KTH-TIPS scale 1). Sub-images of size 128×128 pixels were then extracted from the center of the re-scaled images to build the image sets. We created two distinct sets for experimentation, a training set consisting of 20 unique texture patches (types *a* and *b*) per material and an evaluation sets comprised of 20 unique texture patches (types *c* and *d*) per class. Note that the texture classes rice1 and rice2 as well as stone1, stone2 and stone3, respectively show minimal visual distinction in textural appearance. As a consequence we removed the texture classes rice2, stone2 and stone3 to improve the interpretability of the experiments, leading to a classification scenario with 25 classes.

### 5.2 Compared feature extraction methods

We compare the proposed SOA-LBP to a set of methods, representative for the four categories of scale- and rotation-invariant methods, as discussed in Section 1. We believe that the conceptual properties used by these methods will allow us to establish a comprehensive overview. The used methods are as follows:

**Table:** 

*Category I.* DT-CWT with Log-Polar Transform (*Log-Polar*[1]).
*Category II.* Dominant Scale (*Dominant Scale*[7]).
*Category III.* Fractal Analysis using Filter Banks (*MFS MR8*[10]) and Intersecting Cortical Model (*ICM*[11]).
*Category IV.* Affine Invariant Regions (*Affine Regions*[19]) and Fisher vector encoding of dense SIFT descriptors (*Dense SIFT*[31]). We also compared the method to a standard, multi-resolution LBP with 3 scales (*LBP*[24]) and the proposed scale-invariant LBP representation of Li et al. (*Li-LBP*[21]).

### 5.3 Evaluation protocol and presentation of results

We implemented the experiments in a scale-constrained cross-validation scheme to accommodate for the rather small size of the KTH-TIPS image set. The scheme is based on two distinct sets for training and evaluation. Images for training were always selected from a fixed scale (the default training scale, see Table 1), while the scales for evaluation varied according to the specific experiment. This approach allows us to study the characteristics of each method in reference to signal scaling at various scale differences.

Cross-validation was then performed by an iterated random selection (consistent among all methods) of subsets from the training set (75%) and the evaluation set (25%). A standard *k*-nearest neighbors classifier was used for classification of features extracted from the specific image subsets. The maximum *k*-value corresponds to the number of images in each class of the training set (at maximum 20). The reported results represent the mean accuracy over all *k*-values, averaged in a scale-constrained cross validation with 100 iterations.

We report statistical significance on a per-figure basis to improve the readability. Two-tailed Wilcoxon rank-sum tests were performed at a significance level α=0.001 to assess the null-hypothesis that the population median of the cross-validation results obtained with the proposed methodology (SOA-LBP) is equal to the medians of all corresponding methods presented in the specific figure. An arrow pointing upwards (↑) indicates that the null-hypothesis could always be rejected and the SOA-LBP performed significantly better as compared to all corresponding methods in the figure. An arrow pointing to the right (→) indicates that the null-hypothesis could not be rejected at least once but no significant difference could be identified. Finally an arrow pointing downwards (↓) indicates that at least one method performed significantly better as compared to the proposed method. Note that the markers of each plot are slightly displaced on the *x*-axis to improve the readability of the error-bars, which represent the standard deviations of the individual cross-validation results.

We present the results based on the CURET image set using asymmetric bar charts (Fig. 12). Each side of a bar represents the classification accuracy of a single experiment. The slope of the bar gives an indication of the scale-invariance of each method. The dashed lines represent the average classification accuracies of both experiments. The arrows indicate statistical significance in relation to the SOA-LBP (e.g. an arrow pointing downwards indicates that the specific method performed significantly worse as compared to the proposed methodology).

### 5.4 Studying the effects of image scaling

**Fig. 9:**
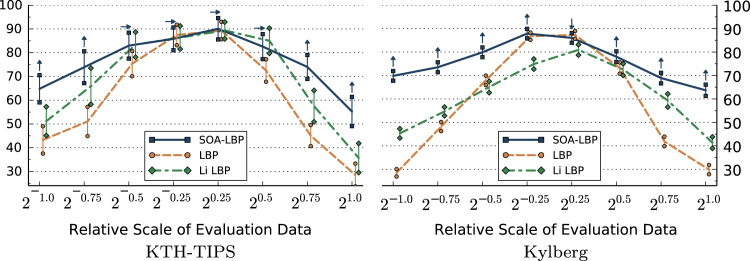
Classification accuracy (*y*-axis) for evaluation scales (scaling only).

**Fig. 10:**
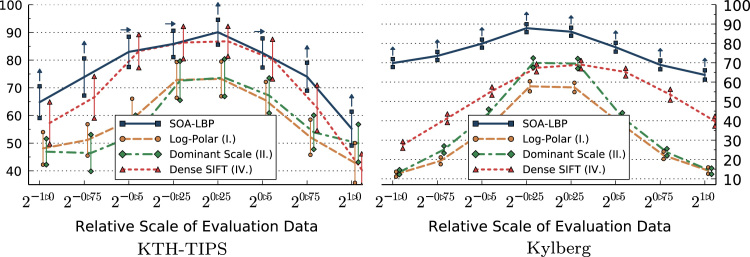
Classification accuracy (*y*-axis) for evaluation scales (scaling only).

**Fig. 11:**
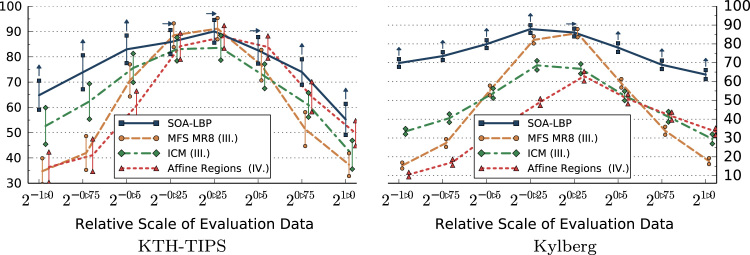
Classification accuracy (*y*-axis) for evaluation scales (scaling only).

**Fig. 12:**
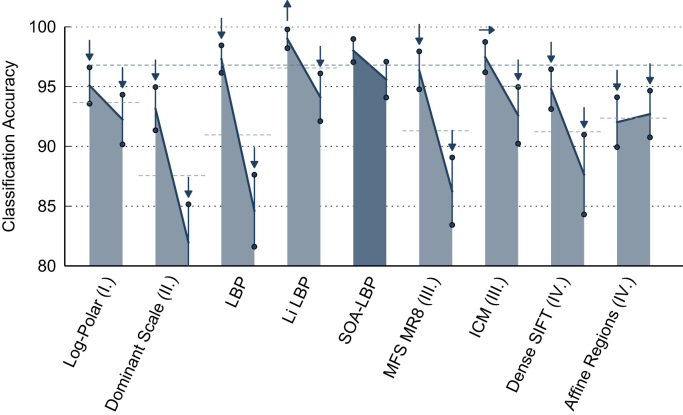
Classification accuracy of the experiments on the CURET data.

The first set of experiments is aimed specifically at studying the characteristics of each evaluated method in regard to image scaling. In these experiments, we only use the scale-invariant representation of methods that allow us a selective use of rotation-invariant features. This includes LBP, Li-LBP, SOA-LBP and Dominant Scale. We present the results of the experiments based on the KTH-TIPS, Kylberg and CURET image sets without rotation in Figs. 9–12. Images at scale $2^{0}$ were used for training, images at all other available scales were used for evaluation (KTH-TIPS and Kylberg).

Based on the CURET data, we follow the experimental setup used by Varma and Zisserman [32]. Two separate training sets were constructed. The first training set consists of textures at both scales, while the second training set is based on textures at a single scale. The evaluation set contains textures at both scales. The difference between the two experiments give an indication for the scale-invariance of each method.

Considering the experiments on the KTH-TIPS image set, we observe that the SOA-LBP performs comparably to the majority of evaluated methods, at evaluation scales close to the training scale. No method performed significantly better as compared to the proposed methodology however, which indicates that the multi-resolution SOA-LBP feature representation is competitive in scenarios with minimal to no scaling. In case of large scale differences (starting at $2^{0.75},2^{-0.75}$) between the training and evaluation data, the SOA-LBP significantly outperforms all evaluated methods.

In parallel to the experiments on the KTH-TIPS data, the SOA-LBPs performance is significantly better as compared to all evaluated methods at large scale differences considering the Kylberg experiments. In contrary to the previous experiments however, this behavior is already recognized at relative scale differences of $2^{0.5}$ and $2^{-0.5}$. The results indicate that the used multi-resolution representation provides highly discriminative features in the more challenging classification problem provided by the Kylberg set, even at tiny scale differences ($2^{0.25}$, $2^{-0.25}$). The only method that performed significantly better as compared to SOA-LBP was the standard multi-resolution LBP at relative scale $2^{0.25}$, which is caused by a small amount of erroneously estimated image scales of the proposed method. Interestingly, the Li-LBP method performed significantly worse even for small scale differences as compared to the standard LBP as well as the proposed method. We assume this characteristic is caused by the direct mapping from estimated scale to the LBP-radius (the average intrinsic-scale of the Kylberg set is higher as compared to the KTH-TIPS data) in combination with a missing, more powerful, multi-resolution representation.

The experiments on the CURET data indicate a high degree of scale-invariance of the SOA-LBP. Only the Li-LBP method performed significantly better in the experiment without required scale-invariance (mixed training scales). The results on the CURET set show that the SOA-LBP is suited for classification in noisy scenarios, outperforming the majority of evaluated methods.

The experiments indicate that the proposed SOA-LBP provides significantly improved classification accuracies in scenarios with large scale differences. The use of intrinsic-scale-adaption allows the computation of discriminative features for a variety of different textures, while the multi-resolution representation provides highly competitive features even in scenarios with tiny scale differences.

### 5.5 Studying the effects of combined image rotation and scaling

**Fig. 13:**
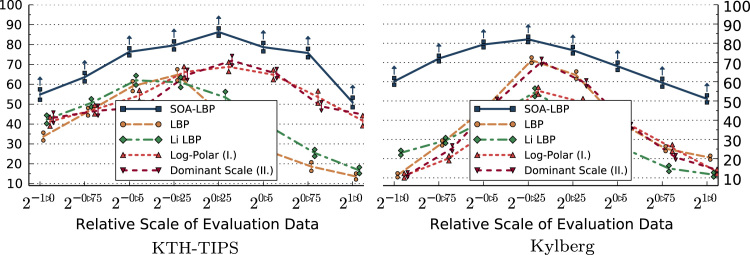
Classification accuracy for evaluation scales (scaling and rotation).

**Fig. 14:**
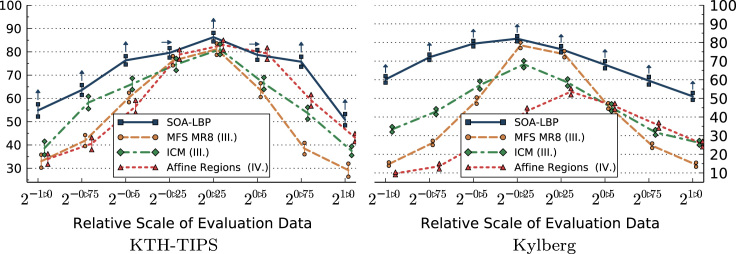
Classification accuracy for evaluation scales (scaling and rotation).

The effects of combined rotation and scaling are studied in the second set of experiments. Feature extraction is based on rotated versions of the Kylberg and the KTH-TIPS image sets. Images at scale $2^{0}$ without rotation were used for training, images at all other available scales were used for evaluation. Subsets of the evaluation sets (KTH-TIPS 891 and Kylberg 1250 images), rotated in steps of 30°, in angles between 30° and 330°, were randomly selected (consistently among all methods) for classification. Only methods providing a scale- and orientation-invariant feature representation were evaluated. LBP was used with the rotation-invariant encoding based on uniform patterns [24]. Li-LBP was used with the proposed sub-uniform patterns [21]. The results are presented in Figs. 13 and 14.

We observe that the rotation of the images decreased the general accuracy of all methods as compared to the previous experiments. The results show the same trends as recognized in the scaling-only experiments however. Again, the proposed SOA-LBP provides significantly improved classification rates at large scale differences between training and evaluation data and performs highly competitive in scenarios with tiny scale differences. The results indicate that the proposed orientation-adaptive computation is superior as compared to encoding-level based approaches used by LBP and Li-LBP. Interestingly, the Li-LBP method performed worse as compared to the standard LBP method on the Kylberg data even at large scale differences. We assume this is caused by the combination of unsuitable LBP-radii (due to the missing intrinsic-scale-adaption) combined with the less discriminative sub-uniform encoding.

The experiments show that the proposed orientation-adapted computation integrates seamlessly into the scale-adaptive LBP. The results are consistent with the previous experiments (scaling only) and indicate that the extraction-level alignment improves the discriminative power of the features.

### 5.6 Runtime performance analysis

**Fig. 15:**
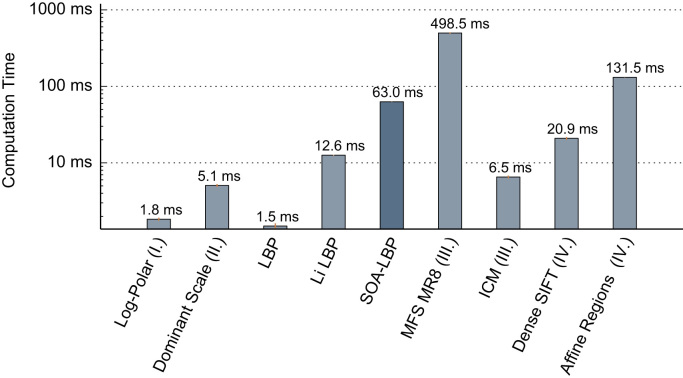
Average computational time per image (KTH-TIPS).

To study the computational demand of the proposed method, we analyze the required runtime of all considered methods in a multi-threaded Java implementation (JDK 8), running on an Intel i5-2500k processor at 4.29 GHz (using a higher frequency multiplier than the default). Due to the nature of the Java programming language (JIT-compilation and garbage collection), we report the computational demand per image as an average of the required computation time for 729 images from the KTH-TIPS data set, in a repeated (20 iterations) experiment (Fig. 15). Note that the presented performance should not be considered an exact benchmark, as not all methods have undergone equal amounts of optimization, but is meant to give the reader an idea of the computational complexity of the proposed methodology.

The results show that the SOA-LBP is considerably slower as compared to the lightweight LBP or the Li-LBP method, which is caused by the increased demand of computing the scales-space, performing scale- and orientation-estimation and the extra amount of feature computation (performing intrinsic-scale-adaption). Considering the improved classification accuracy in environments with varying scales and orientations however, we think that the average computational demand of 63 ms per image is an adequate trade-off. This is even emphasized as the method ranks in the lower middle range among all methods.

## 6 Conclusion

We presented a generic methodology to compute a scale- and rotation-invariant feature representation based on LBP, by suitable adaption of the LBP neighborhood. The use of intrinsic-scale-adaption allowed the computation of features, independent of the intrinsic-scale of textures, and increased the reliability of the method significantly. This has been shown in experiments based on three different image sets representing a variety of scenarios. The SOA-LBP was significantly superior to all evaluated methods in case of large scale differences. The proposed multi-resolution feature representation was more than competitive in scenarios with tiny scale differences. Experimentation based on the noisy CURET data showed that the proposed methodology provides discriminative and reliable features in difficult scenarios.

Although the computational complexity of the SOA-LBP is significantly higher as compared to the very lightweight LBP, we regard the improved classification accuracies in scenarios with scaling and rotation as an acceptable trade-off for many classification tasks. The proposed methodology is easily applied to a wide variety of LBP based methods [26,27], providing a robust scale- and rotation-invariant feature representation.

## Conflict of interest

None declared.

## Figures and Tables

**Table 1:** Information on the image sets used for experimentation.

**Database**	**Classes**	**Images per scale**	**Scales**	**Training scale**
CURET	4	184	2	Mixed
KTH-TIPS	9	81	9	$2^{0}$
Kylberg	25	500	9	$2^{0}$
